# Personality Traits and Training Initiation Process: Intention, Planning, and Action Initiation

**DOI:** 10.3389/fpsyg.2016.01767

**Published:** 2016-11-17

**Authors:** Mariola Laguna, Ewelina Purc

**Affiliations:** Institute of Psychology, The John Paul II Catholic University of LublinLublin, Poland

**Keywords:** personality traits, training, intention, planning, goals

## Abstract

The article aims at investigating the role of personality traits in relation to training initiation. Training initiation is conceptualized as a goal realization process, and explained using goal theories. There are three stages of the process analyzed: intention to undertake training, plan formulation, and actual training undertaking. Two studies tested the relationships between five personality traits, defined according to the five factor model, and the stages of the goal realization process. In Study 1, which explains training intention and training plans’ formulation, 155 employees participated. In Study 2, which was time-lagged with two measurement points, and which explains intention, plans, and training actions undertaken, the data from 176 employees was collected at 3 month intervals. The results of these studies show that personality traits, mainly openness to experience, predict the training initiation process to some degree: intention, plans, and actual action initiation. The findings allow us to provide recommendations for practitioners responsible for human resource development. The assessment of openness to experience in employees helps predict their motivation to participate in training activities. To increase training motivation it is vital to strengthen intentions to undertake training, and to encourage training action planning.

## Introduction

These days, many people aim at improving their professional skills and acquiring new competences through training. Ideas of lifelong learning and personal development are more and more popular (European Commission, 2012). Formal education appears insufficient for obtaining all the competences necessary to function in today’s dynamic economy. It is especially important for employees, who not only want, but are often forced, to develop their competences, otherwise they will not remain in the demanding labor market (Brown and Lauder, 1996; Willyerd and Mistick, 2016). There is, however, no easy answer to the question as to why one person takes up training activities and another does not, as well as which personal characteristics foster training initiation. As the results of previous research are not coherent, as we show in subsequent sections, there is a vital need for more research explaining training initiation.

Previous studies focused primarily on training methods, in an attempt to identify factors that increase training effectiveness (e.g., Arthur et al., 2003; Banerjee et al., 2016). However, before starting to learn by means of a variety of methods, one must first be motivated to initiate training. Although popular conceptions regarding the process of learning in adults (e.g., Knowles, 1980; Kolb, 1984) emphasize that learners bring their own characteristics to the learning process and may have different motivations to learn, these issues have relatively rarely been the subject of empirical research. Due to the small number of empirical studies indicating which personality factors and which situational factors are important to training, only one meta-analysis has been published to date (Colquitt et al., 2000). It shows that personality traits play a role in training undertaking and training effectiveness. The authors, however, point to a clear need for further research and for developing theoretical concepts. There is some evidence showing that individual differences, such as proactive personality or self-efficacy, play important roles in training motivation (Warr and Birdi, 1998; Tracey et al., 2001; Major et al., 2006; Bertolino et al., 2011). Although personality has been examined as related to training motivation (Godlewska-Werner et al., 2014), knowledge about these relationships is still insufficient. Studies concerning personality traits described by the widely acknowledged five factor model (McCrae and Costa, 1999) are rare and do not offer a definitive answer, although this issue is important both theoretically and practically.

Therefore, the studies presented in this article aim at investigating the relationships between five personality traits and training initiation. We also propose a theoretical model of training initiation that meets the need for the development of theoretical concepts in this area of study (Colquitt et al., 2000; Salas and Cannon-Bowers, 2001). In this way, the article offers a new insight into the relationships between personality traits and training initiation, as it (1) proposes a new model of training initiation, (2) investigates the role of five personality traits, and (3) tests the models in two independent studies. To explain our theoretical framework, we firstly present the concept of training initiation as a goal realization process (Heckhausen and Gollwitzer, 1987; Gollwitzer, 1999); secondly, we explain the role of personality traits in training initiation.

### Undertaking Training as a Goal Realization Process

Training and competence development produces beneficial effects both for an organization and an employee. Modern organizations make efforts to continuously improve their employees’ skills by implementing human resource development strategies. Effective training increases efficiency and allows an organization to gain a competitive advantage over other organizations (Salas and Cannon-Bowers, 2001). At the same time, the tendency to delegate responsibility for one’s own development from the organization to the individual employee is becoming more and more apparent (Willyerd and Mistick, 2016). Changes in the labor market, as well as a growing tendency to pursue a self-managed career, ‘protean careers’ and ‘boundaryless careers’ (Inkson, 2006) forces people to be more independent in searching for training and opportunities to increase their competence to remain in the dynamic labor market and find satisfactory employment (Major et al., 2006; Willyerd and Mistick, 2016). This makes training initiation an important issue that needs careful analysis.

Recent years have seen an increase in the availability and flexibility of different forms of professional development (e.g., European Commission, 2012). Various forms of training have become much more accessible, often free of charge (Czernecka et al., 2011). Despite the increase in accessibility, only a relatively small percentage of adults endeavor to improve their qualifications, even though the income of people who take part in training increases considerably compared to the income of those who have not taken such actions (Czapiñski and Panek, 2011). Therefore, it is important to ask what factors favor participation in training and development activities. Motivation to initiate training is of great significance to taking actual action; it is an important predictor of attitudes toward training (Carlson et al., 2000), of actually undertaking training (Tharenou, 2001) and also of the transfer of knowledge and skills to everyday situations at work (Baldwin and Ford, 1988; Gegenfurtner and Vauras, 2012). Training motivation depends on both individual and situational factors, as revealed by the meta-analysis that included 106 studies (Colquitt et al., 2000). However, this meta-analysis covered only some of the relationships among personality factors, job- and career-related factors, situational factors, motivation to learn, and the results of the learning process. Although the initial theoretical model included a broad set of variables, the final meta-analysis could include only a few determinants of motivation because of the insufficient number of existing studies which did not allow other variables to be included in the meta-analysis. Meta-analytic path analysis showed that motivation to learn can be explained by personality, age and positive organizational climate (Colquitt et al., 2000). Other studies attesting to organizational determinants proved that training motivation is positively related with organizational commitment (Naquin and Holton, 2002; Cunningham and Mahoney, 2004), perceived organizational justice (Kang, 2007), distributive justice, and utility (Bell and Ford, 2007).

Many of the previous studies, however, are rather descriptive and atheoretical; therefore, it is necessary to develop theories that explain the relations between personality and situational factors and training initiation (Colquitt et al., 2000). Embedding the research on training motivation within a broader theoretical context would allow a broader interpretation of the research results. A model of the goal realization process could serve this purpose and has already been successfully used in other studies (Brandstätter et al., 2003; Godlewska-Werner et al., 2014; Łaguna et al., 2015a,b; Mielniczuk and Laguna, in press).

In order to analyze the motivational mechanisms that lead to taking self-development actions, we cannot treat them as a single acts but rather as a process, the final phase of which is actually taking action. Participation in training may be treated as a goal-directed behavior. Similarly to other behaviors (e.g., voting in an election, Ajzen, 1991; starting up a new business, Laguna, 2013), participation in training may be considered as purposive behavior that is planned in advance (Heckhausen and Gollwitzer, 1987; Brandstätter et al., 2003). Treating it as a goal realization process allows an analysis of the mental processes that precede taking action, such as formulating intentions to achieve the goal, and action planning. It also allows the analysis of personal characteristics in relation to the successive stages of this process.

The model of goal realization process describes four phases: predecisional, preactional, actional, and postactional (Heckhausen and Gollwitzer, 1987). We concentrate on the first two phases, which precede action initiation. At the predecisional phase, a positive subjective evaluation of the goal may lead to the formulation of an intention to attain it, which is an important determinant for taking action. The stronger the intention to undertake specific behavior (behavioral intention), the greater the likelihood is of its effective realization (Armitage and Conner, 2001; Hurtz and Williams, 2009). Developing behavioral intention starts the next preactional phase. Research shows, however, that intention alone is not always sufficient (Gollwitzer et al., 2008; Gollwitzer and Oettingen, 2012). With the intention to undertake training, at the preactional phase, a person should also plan where, when, and what developmental activities they want to undertake in order to attain the goal. Action planning, also called the formulation of implementation intentions, is a significant antecedent of successful goal realization (Gollwitzer, 1999) as has been shown in many studies (e.g., Brandstätter et al., 2003; Loy et al., 2016). In the next step, the actional phase, in order for a person to attain a goal, they must take actual action and effectively complete it. After achieving the goal, the postactional phase occurs, in which the doer evaluates the results and consequences of the achieved goal. It is also possible to stop at each of these stages and to not initiate activity.

Using the model of action phases (Heckhausen and Gollwitzer, 1987; Gollwitzer, 1999) it is possible to analyze intention to undertake training (which can be expressed as a declaration, such as, *I want to start the training*), plans to initiate training activities (i.e., where, when, what kind of training to choose), and training action initiation phase (e.g., enrolling in training, starting a course). A lack of intention or of clear action plans may be responsible for not taking up training, since research results concerning other activities show that behavioral intentions and action plans are closely related (for a review, see Gollwitzer, 1999; Gollwitzer and Oettingen, 2012). According to this model, goal intention is related to action planning, and planning is related to the initiation of action that leads to goal realization. Therefore, we might expect that:

(1) *Hypothesis 1*: Intention to undertake training will be positively related to planning this activity (*Hypothesis 1a*), and training action initiation (*Hypothesis 1b*).
(1) *Hypothesis 2*: Training action planning will be positively related to training action initiation.
(1) *Hypothesis 3*: Training action planning will mediate between the intention to undertake training and training action initiation.

### Personality Traits and Training Initiation

Determining personal variables that are important at the stages preceding action initiation may help us understand why some people undertake training, and others do not. Personality traits are considered basic personal characteristics that may be favorable to the initiation of the motivational processes of goal pursuit (Judge and Llies, 2002). The widely adopted five factor model of personality (McCrae and Costa, 1999) identifies five main dimensions: extraversion (i.e., ease in social interactions, preference for being in groups, tendency to be active, talkativeness); agreeableness (i.e., the tendency to trust, altruism, cooperation); conscientiousness (i.e., the tendency to be purposeful, ambitious, reliable, organized, and determined); openness to experience (i.e., intellectual curiosity, preference for novelty, imagination), and neuroticism (i.e., vulnerability to the experience of unpleasant emotions, difficulty in coping with stress), which is the opposite of emotional stability.

The five factor model is intensively studied in various areas of psychology (McCrae and Costa, 1999), and personality traits are considered to be important sources of motivation (Judge and Llies, 2002). Research shows that they are related to performance motivation (Judge and Llies, 2002), as well as to academic motivation and achievement (Komarraju et al., 2009, 2011). These basic individual characteristics may also play a role in training initiation. According to the theory (McCrae and Costa, 1999), extraverted people, being sociable, active, and eager to engage in social interactions, are probably willing to undertake activities, such as training, which usually demand interactions with others. Also agreeableness may be related to undertaking training, because it is manifested by good cooperation with people. Conscientiousness may be important for training motivation, as someone who is conscientious, perseveres in goal realization. Openness to experience may show a positive relationship with undertaking training, as training offers new challenges, activities and new experiences. Neuroticism may be unfavorable for goal realization, as it is accompanied by negative affect and poor coping strategies (McCrae and Costa, 1999).

However, there are few studies investigating the link between personality traits and engagement in professional development. In empirical research, the most frequently examined trait was conscientiousness. Colquitt and Simmering (1998) analyzed its links with motivation to learn. Students participated in a six-week course; after two weeks they received feedback concerning the extent to which they had accomplished their learning goals. Both motivation to learn measured before the course (initial motivation) and post-feedback motivation correlated significantly with conscientiousness. In other studies, conscientiousness was found to be correlated with training motivation (Morrell and Korsgaard, 2011) and training outcomes (Vasilopoulos et al., 2007), but it was not a significant predictor of actually engaging in training (Hurtz and Williams, 2009). Openness to change was positively related with intention to participate in training and with planning to undertake training (Godlewska-Werner et al., 2014). Conscientiousness was the only one of the five personality dimensions that was taken into account in the meta-analysis of training motivation determinants (Colquitt et al., 2000). In the meta-analysis of single correlations, conscientiousness was significantly correlated with motivation to learn (*r* = 0.38). At the same time, in the meta-analytic paths analysis, the path coefficient for the conscientiousness–training motivation relationship was only -0.01 and not statistically significant. Moreover, these results are not conclusive as they are based on a relatively small (especially for a meta-analysis) sample of 550 people. The authors indicate the need for further studies that will provide more data and recommend the investigation of other dimensions of the five factor model, which were studied so rarely that they could not be incorporated into the meta-analysis (Colquitt et al., 2000).

Indeed, studies taking all five personality traits into account are rare. For example, Rowold (2007) showed that the higher the level of introversion, the lower the motivation to learn. Agreeableness and openness to experience correlate with motivation to learn, both significantly and positively, while emotional instability (the counterpart of neuroticism) and motivation to learn were uncorrelated. Conscientiousness was also not significantly correlated with motivation to learn. In another study, Major et al. (2006) showed that extraversion, openness to experience, and conscientiousness are positively correlated, while neuroticism and agreeableness are uncorrelated with motivation to learn.

The results of the few studies carried out to date are not fully conclusive, especially with regards to agreeableness and neuroticism. This indicates the need for further studies and the necessity of replicating the results in different samples. Previous analyses also confirmed that all personality traits, not just conscientiousness, should be included in further studies (Major et al., 2006; Rowold, 2007). Due to the inconclusive nature of previous research findings, we pose the following research question:

*Research question 1*: What are the relationships between the five personality traits and training initiation: intention, plan and action initiation?

It is proposed in previous studies (Colquitt et al., 2000) that personality traits may affect motivation to participate in training directly or indirectly through other variables. The model with direct relationships was better supported by the data (Colquitt et al., 2000), however, it was postulated that further research is needed to attest to those relationships. In our studies, based on the model of the goal realization process (Heckhausen and Gollwitzer, 1987), we propose that behavioral intention serves as a mediator between personality traits (McCrae and Costa, 1999) and further stages of the goal realization process. We hypothesize that:

(1) *Hypothesis 4:* Intention to initiate training mediates between personality traits and training action planning (*Hypothesis 4a*) and training action initiation (*Hypothesis 4b*).

### Current Studies

In order to test the role of personality traits in relation to training initiation understood as a goal realization process, two studies were carried out. They filled a gap in the existing state of knowledge, because they: (1) took into account all the five dimensions of personality, thereby giving a more comprehensive picture; (2) referred to the theoretical model of training initiation as a goal realization process, and (3) tested the models on two independent samples, using a longitudinal study design in Study 2.

## Study 1

The aim of this study was to investigate the relationships between personality traits and two phases of training initiation process: training intention formulation and training action planning.

### Method

#### Procedure

The participants, in individual contact with researchers, received paper-and-pencil questionnaires to complete. After completing the questionnaires they placed them into sealed envelopes, and the researcher picked them up the following day. Participation in the study was voluntary and unpaid, and the participants were guaranteed anonymity.

#### Participants

The study participants were 155 employees of small companies (from 10 to 50 employees), including 79 women (51%), aged from 20 to 65 years (*M* = 37.4, *SD* = 10.17). Almost half of them (47.7%) had completed higher education, 38.2% had secondary education, and 12.9% had vocational education. Most of the participants (98, 63.2%), were employed on a permanent basis, 37 participants (24%) were employed temporarily, and 19 (12.2%) had other types of contracts.

#### Measures

*Personality traits* were measured using the 10-Item Personality Inventory (Gosling et al., 2003; Łaguna et al., 2014). It begins with the phrase *I see myself as*, followed by 10 pairs of adjectives (e.g., *extraverted, enthusiastic*). The answers are provided on a seven-point scale, from 1 – *strongly disagree* to 7 – *strongly agree*. The score for each of the five personality dimensions is the mean score of two statements, higher scores indicate stronger intensity of a trait. Test-retest reliability (recommended as a good indicator of the reliability of short scales, Gosling et al., 2003) with a 2-week interval was 0.62 for openness to experience, 0.70 for emotional stability, 0.71 for conscientiousness, 0.74 for agreeableness, and 0.77 for extraversion. The validity of this widely used instrument has been confirmed in many studies (Gosling et al., 2003; Łaguna et al., 2014).

To measure *training intention*, the Training Intention Scale (Kawecka et al., 2010) was used. The scale consists of three items (a sample item: *I would like to undertake training*) which are answered on a five-point scale from 1 – *definitely not* to 5 – *definitely yes*. Higher scores indicate higher levels of the variable. The reliability of this study indicated by Cronbach’s α was 0.92. Confirmatory factor analysis (CFA) confirmed that the scale captured single latent factor (Kawecka et al., 2010).

To assess *training action planning* the Training Planning Scale (Kawecka et al., 2010) was applied. It consists of three items (a sample item: *I have already decided where I will undertake training*), the answers are provided on a five-point scale from 1 – *definitely not* to 5 – *definitely yes*. The higher the sum of these items, the higher is the level of the variable. Cronbach’s α reliability in this study was 0.84. Single latent factor assessed by the scale was confirmed by CFA (Kawecka et al., 2010).

#### Data analysis

In order to analyze multivariate relationships, hierarchical regression analysis was used. Because in previous studies demographic variables reported as salient to training motivation (Major et al., 2006; Bertolino et al., 2011), they were included in the analyses. In the subsequent models first sex and age, and then the five personality traits were entered as predictors of intention to undertake training (Models 1 and 2) and of action planning (Models 3 and 4). In the last model predicting action planning (Model 5), training intention was added.

Finally, mediation analysis was performed to test the mediation hypothesis (*H3a*), following Hayes (2013) recommendations. The PROCESS macro (Hayes, 2013) was employed for attesting to the mediation effect. This procedure estimates an indirect effect using a bootstrapping technique. Bias-corrected and accelerated bootstrapping was conducted in the present analyses using 5,000 repetitions. For each mediation analysis, an indirect effect, together with bootstrapped standard error (*SEB*) and 95% confidence interval (CI), will be reported. If the confidence interval does not include zero, it confirms a statistically significant mediation effect.

To check the potential common method bias, the Harman’s single factor test was used as the simplest and most effective test of common method variance (CMV; Fuller et al., 2016). We loaded all items of all measures used in the study into an exploratory factor analysis and examined the unrotated solution. A single factor which accounted for the majority of the covariance among measures did not emerge. We have obtained a five factor solution with the first factor explaining 29.23% of variance. As the first factor does not account for more than 50–60% of the variance among variables (Fuller et al., 2016), the research results do not suffer from the CMV problem.

### Results

The results of the single correlation analysis (**Table 1**) indicate that personality traits are generally correlated more with training intention than with planning of action. Personality traits which show statistically significant positive correlations with training intention are openness to experience, emotional stability (the opposite of neuroticism), extraversion, and agreeableness while, with training plans formulation, it is openness to experience and extraversion. Conscientiousness does not show statistically significant correlations with any of these motivational variables. Moderate correlations between personality traits suggest there was no serious multicollinearity problem. This was further confirmed by the variance inflation factor (VIF); VIF scores ranged from 1.01 to 1.46. Correlation between intention and planning is statistically significant, positive, and relatively high, which confirms *Hypothesis 1a*.

**Table 1:** Descriptive statistics and correlations between variables in Study 1 (*N* = 155).

Variables	Scores ranges	*M*	*SD*	1	2	3	4	5	6
(1) Extraversion	1–7	4.65	1.15						
(2) Agreeableness	1–7	5.22	1.15	0.01					
(3) Conscientiousness	1–7	5.51	1.30	0.12	0.37^∗∗∗^				
(4) Openness to experience	1–7	5.27	1.23	0.44^∗∗∗^	0.24^∗∗^	0.39^∗∗∗^			
(5) Emotional stability	1–7	4.70	1.28	0.06	0.37^∗∗∗^	0.21^∗∗^	0.31^∗∗∗^		
(6) Training intention	3–15	10.29	3.16	0.18^∗^	0.18^∗^	0.10	0.41^∗∗∗^	0.29^∗∗∗^	
(7) Training planning	3–15	8.39	2.97	0.16^∗^	0.02	-0.04	0.29^∗∗∗^	0.12	0.52^∗∗∗^


*Pearson *r* correlations are reported; ^∗∗∗^*p* < 0.001, ^∗∗^*p* < 0.01, ^∗^*p* < 0.05 (two-tailed).*

Two models explaining the intention to undertake training were tested using hierarchical multivariate regression analysis. In the first model, sex and age were entered as predictors (Model 1). Only age proved to be statistically significant, showing that training intention decreases with age (**Table 2**). In Model 2, personality traits were added to the model. This analysis shows that openness to experience is the only statistically significant predictor of training intention. According to these findings, the higher the level of openness to experience, the higher the intention to participate in training. Other personality traits do not show significant relationships with training intention. The variables included in the model explain 22% of variance in training intention.

**Table 2:** Results of hierarchical multiple regression analyses predicting training intention (Models 1 and 2) and training planning (Models 3–5).

Predictors	Dependent variable				
	
	Training intention		Training planning		
		
	Model 1	Model 2	Model 3	Model 4	Model 5
Sex	-0.03	0.01	-0.03	0.00	-0.00
Age	-3.51***	-0.24**	-0.08	-0.02	0.10
Extraversion		-0.02		0.05	0.06
Agreeableness		-0.02		-0.02	-0.06
Conscientiousness		-0.09		-0.08	-0.06
Openness to experience		0.35***		0.34***	0.18+
Emotional stability		0.13		0.03	-0.03
Training intention					0.48***
Model fit					
*F*	6.23**	6.60***	0.48	2.76**	7.06***
*R*^2^	0.07	0.22	0.01	0.08	0.26
Δ*R*^2^	0.08**	0.17***	0.01	0.12**	0.17***


*Standardized β regression coefficients are reported for each model; ^∗∗∗^*p* < 0.001, ^∗∗^*p* < 0.01, ^+^*p* < 0.10.*

In the model explaining action planning (Model 3), none of the demographic variables occurred as statistically significant. After adding five personality traits into the regression equation (Model 4), only openness to experience proved to be a statistically significant predictor of training action planning. In the last step, training intention was added into the model (Model 5), occurring as a significant predictor of planning, further confirming *Hypothesis 1a*. All variables included in the final model explain 26% of variance in action planning.

A mediation analysis using the PROCESS macro (Hayes, 2013) was performed to test the hypothesis that the intention to initiate training mediates between personality traits and training action planning (*Hypothesis 4a*). It showed a significant indirect effect of four personality traits via training intention to training plans: extraversion (*B* = 0.25, *SEB* = 0.12, 95% CI [0.03; 0.51]); agreeableness (*B* = 0.26, *SEB* = 0.14, 95% CI [0.03; 0.56]); openness to experience (*B* = 0.48, *SEB* = 0.13, 95% CI [0.26; 0.76]), and emotional stability (*B* = 0.34, *SEB* = 0.12, 95% CI [0.13; 0.61]), as each of 95% confidence intervals did not include zero. Only where conscientiousness was concerned, was the indirect effect of training intention insignificant (*B* = 0.11, *SEB* = 0.10, 95% CI [-0.08; 0.33]). To conclude, mediation analyses partially confirm *Hypothesis 4a*, except for conscientiousness.

### Discussion

The results of this study indicate the important role of openness to experience. This variable was previously rarely included in studies. Moreover, few findings demonstrated links between openness to experience and training motivation (Rowold, 2007; Godlewska-Werner et al., 2014) or training performance (Lievens et al., 2003), while others did not show any such relationships (Naquin and Holton, 2002). This study confirms that openness to experience is important to the training initiation process. People who are broad-minded, curious, willing to experience new situations, and to meet new people are more ready to form intentions to participate in training. Moreover, they more often plan when, where and what training they want to start. The results also confirm clear links between the intention to undertake training and training action planning. This relationship is also acknowledged in the results of studies on other purposive behaviors (Gollwitzer et al., 2008; Gollwitzer and Oettingen, 2012).

Other personality traits (extraversion, agreeableness, conscientiousness, and emotional stability), seemed to be not related to training intention and training action planning in the regression analysis. Nevertheless, some of them proved to be indirectly related to training plans. In the classic theory of mediation by Baron and Kenny (1986) it was not advisable to analyze mediation when independent and dependent variables were not significantly related to each other. However, in the modern approach proposed by Hayes (2009, 2013), we can analyze indirect effects even when the direct relationship is insignificant. Mediation analyses showed that training intention is a mediator in relationships between four personality traits (extraversion, agreeableness, openness to experience, emotional stability) and planning of training action. It supports including mediating mechanisms between more general and distal personality characteristics and action (Rauch and Frese, 2007).

The fact that one of the last stages of the goal realization process – taking actual action – is not included in this study constitutes its limitation as it does not allow us to assess the role of personality traits in this phase of the process. Another limitation results from the use of the short 10-item TIPI measure (Gosling et al., 2003), which may lead to an underestimation of the role of personality traits, even though TIPI shows good psychometric properties compared to other very short measures (Gosling et al., 2003; Credé et al., 2012).

## Study 2

In order to check the generalizability of the results of Study 1, a second study was carried out. It included not only the formulation of training intentions and plans, but also actual training initiation. Moreover, it applied another measure of personality traits, proving the generalization of results from Study 1.

### Method

#### Procedure

A time-lagged design with two time points was used (Łaguna, 2012). The participants, in individual contact with trained researchers, filled in all measures in a paper and pencil format and, after 3 months, were contacted personally to answer questions regarding the training activities they had actually undertaken and completed. Such a time interval allows us to take actual actions into account as well as to observe the relationships between study variables and taking action (Ajzen, 1991; Laguna, 2013). Participation in the study was voluntary, without any reward, and the participants were guaranteed confidentiality.

#### Participants

The study involved 209 employees from small and medium-sized companies (from 10 to 250 employees), of whom 176 (84.2% of the sample) also answered questions after 3 months. The second study wave respondents did not differ from non-respondents in any of the study variables measured at the first stage of data gathering. Only the data from participants who took part in both study waves were taken into account in the further analysis. The final sample included 80 men and 96 women, aged from 20 to 57 (the mean age was 38.41 years, *SD* = 10.31). As regards education, 106 participants (60.2%) had completed higher education, 51 (29.0%) had secondary education, and 19 (10.8%) had vocational education. Most of them were employed on a permanent basis (137 participants, 77.8%), some had temporary employment (35 participants, 19.9%), and four did not provide this information.

#### Measures

To measure *personality traits*, the NEO-FFI (Costa and McCrae, 1992) was used (Zawadzki et al., 1998). It consists of 60 statements to which responses are provided on a five-point scale from 1 – *strongly disagree* to 5 – *strongly agree*. The higher the score for each scale the higher the level of that trait. Cronbach’s α reliability ranges from 0.68 for openness to experience, 0.78 for agreeableness, 0.79 for neuroticism, 0.80 for conscientiousness, to 0.82 for extraversion.

*Training intention* and *training action planning* were measured with the Training Intention Scale and the Training Planning Scale (Kawecka et al., 2010), the same as in Study 1. The reliability in this study was α = 0.91 and α = 0.88 for intention and planning, respectively.

After 3 months, the participants were asked to answer questions regarding *training actions* taken during that time. The response scale contained four options: 1 – *I did nothing to undertake training*; 2 – *I signed up and I am waiting for training to begin*; 3 – *I have started training*; 4 – *I have already taken up and finished training*.

#### Data Analysis

Similarly to Study 1, a hierarchical multivariate regression analysis was used. In the first five models explaining the intention to undertake training and training action planning, a hierarchical linear regression was used. In the next four models predicting training action initiation, a multiple hierarchical logistic regression analysis was applied. This enabled us to predict the presence or absence of a training action initiation on the basis of subsequent sets of predictors (Norušis, 1999). Mediation analysis using the PROCESS macro (Hayes, 2013) with bootstrapping using 5,000 repetitions was performed to attest to *Hypothesis 3* and *Hypothesis 4*.

As in Study 1, the Harman’s single factor test (Fuller et al., 2016) was used to check the CMV. An exploratory factor analysis on all items from all measures did not show the single factor solution. The 20 factor solution emerged with the first factor explaining 11.35% of variance. Thus, the common method bias did not affect the study results considerably.

### Results

The analysis of single correlations (**Table 3**) shows that there are statistically significant positive relationships between personality traits and intention to undertake training, except of neuroticism. Only conscientiousness and openness to experience are statistically significantly correlated with training action planning, while none of the correlations of personality traits with the training action initiation is statistically significant. Correlations between training intention and action planning is positive and statistically significant, which confirms Hypothesis 1a. The actual taking of action after three months is significantly correlated with the intention to undertake training and with planning, which confirms Hypothesis 1b and Hypothesis 1c. Intercorrelations between personality traits are moderate (from -0.08 to -0.34), and only some of them are statistically significant. This suggests no serious multicollinearity problem, further confirmed by the VIF scores, which ranged from 1.02 to 1.36.

**Table 3:** Descriptive statistics and correlations between variables in Study 2 (*N* = 209).

Variables	Scores ranges	*M*	*SD*	1	2	3	4	5	6	7
(1) Extraversion	12–60	36.45	6.64							
(2) Agreeableness	12–60	29.87	5.20	0.27***						
(3) Conscientiousness	12–60	33.63	6.08	0.30***	0.32***					
(4) Openness to experience	12–60	24.61	5.46	0.32***	0.03	0.11				
(5) Neuroticism	12–60	20.30	7.02	-0.21**	-0.34***	-0.19*	-0.08			
(6) Training intention	3–15	11.17	3.09	0.20**	0.17*	0.21**	0.25***	-0.07		
(7) Training planning	3–15	8.48	3.58	0.13	0.04	0.17*	0.18*	-0.12	0.55***	
(8) Training action^a^	1–4	1.67	1.05	0.02	0.05	0.04	0.18*	0.08	0.19*	0.29***


*Pearson *r* correlations are reported; ^a^ for ‘Training Action’ Spearman *rho* correlations are reported; ^∗∗∗^*p* < 0.001, ^∗∗^*p* < 0.01, ^∗^*p* < 0.05 (two-tailed).*

Despite a relatively high mean level of intention to undertake training (*M* = 11.17 on a scale from 3 to 15, see **Table 3**), the majority of participants did not take real action (117 participants, 66.5%). A relatively small group had registered for training and was waiting for it to begin (18 participants, 10.2%), 23 participants (13.1%) were engaged in training at that time, and 18 (10.2%) had already completed it. Therefore, a total of 33.5% of the sample did take any action aimed at training initiation. Based on these results the variable ‘Training action’ was dichotomized before entering into further analyses and coded 1 – *initiation of any action leading to training completion* (33.5% of the sample), and 0 – *no action initiated* (66.5% of the sample).

A hierarchical multivariate regression analysis was used in the models explaining training intention (Models 1 and 2, **Table 4**), as well as in the models explaining training action planning (Models 3–5). In the first model (Model 1), in which sex and age were entered as predictors of the intention to undertake training, only age proved to be statistically significant. It also remained significant after entering the five personality traits into the equation (Model 2). The older a person is, the lower their training intention. In Model 2, only openness to experience occurred as a statistically significant predictor, while other personality traits did not. A higher level of openness to experience favors a higher intention to undertake training. Demographic variables, together with personality traits, explain 14% of the variance in training intention.

**Table 4:** Results of hierarchical multiple regression analyses predicting training intention (Models 1 and 2), training planning (Models 3–5), and training action initiation (Models 6–9, logistic regression results).

Predictors	Dependent variable								
	
	Training intention		Training planning			Training action			
			
	Model 1	Model 2	Model 3	Model 4	Model 5	Model 6	Model 7	Model 8	Model 9
Sex	0.14+	0.07	0.05	0.03	-0.01	0.66^+^ (1.93)	0.55 (1.72)	0.50 (1.65)	0.53 (1.69)
Age	-0.26***	-0.22**	-0.22**	-0.18*	-0.06	-0.03^∗^ (0.97)	-0.03^∗^ (0.97)	-0.03 (0.97)	-0.02 (0.97)
Extraversion		0.01		-0.01	-0.02		-0.03 (0.97)	-0.03 (0.98)	-0.03 (0.98)
Agreeableness		0.09		0.01	-0.03		0.04 (1.04)	0.04 (1.04)	0.04 (1.04)
Conscientiousness		0.09		0.10	0.05		0.00 (1.00)	-0.01 (0.99)	-0.01 (0.99)
Openness to experience		0.24**		0.15*	0.04		0.07^∗^ (1.07)	0.06^+^ (1.06)	0.06^+^ (1.06)
Neuroticism		-0.00		-0.07	-0.07		0.01 (1.02)	0.01 (1.01)	0.02 (1.02)
Training intention					0.51***			0.12^+^ (1.13)	0.02 (1.03)
Training planning									0.16^∗∗^ (1.17)
Model fit	9.90***	5.58***	5.20**	2.78**	10.16***	6.93	6.75	4.45	5.20
*R*^2^	0.08	0.14	0.04	0.06	0.28	0.07	0.11	0.14	0.19
Δ*R*^2^	0.09***	0.08**	0.05**	0.04	0.21***	–	–	–	–


*Standardized β regression coefficients are reported for Models 1–5, and B together with Exp(B) (in brackets) for Models 6–9; ^∗∗∗^*p* < 0.001, ^∗∗^*p* < 0.01, ^∗^*p* < 0.05, ^+^*p* < 0.10; as model fit indicators for Models 1–5 *F* and *R*^2^ statistic are reported, for Models 6–9 chi^2^ of Hosmer’s and Lemeshow’s test (*df* = 8) and Negelkerk’s R^2^ are reported.*

The next three models explain planning of training actions. Again, age proved to be negatively related to training plans, even after entering personality traits into the equation (Models 3 and 4). Of the five personality traits (Model 4), only openness to experience significantly predicts the level of training plans. Training intention entered into the model (Model 5) revealed a significant predictor. The higher the training intention, the higher the action planning. All variables included in the final model explain 28% of variance in training action planning.

Because the training action initiation was treated as a dichotomous variable (the participant initiated or did not initiate action after three months), hierarchical logistic regression was applied in models explaining training action initiation (Models 6–9; **Table 4**). In the first of these models (Model 6), age appeared negatively related to action initiation. After including personality traits (Model 7), again, only openness to experience occurred as an important predictor of training action initiation. Training intention entered in Model 8 did not reach a level of statistical significance. Training action planning (Model 9) was proved to significantly predict training action initiation.

*Hypothesis 3* was attested by mediation analysis. Regression analyses confirmed (**Table 4**), that training intention is good predictor of training action planning, and planning, in turn, predicts training action initiation. Mediation analysis using the PROCESS macro (Hayes, 2013) showed an insignificant indirect effect of the training intention via training plans to training action initiation (*B* = 0.07, *SEB* = 0.34, 95% CI [-0.30; 0.63]). As the 95% confidence interval includes zero, it does not confirm *Hypothesis 3*.

To attest to *Hypothesis 4*, two mediation analyses were performed. The first one attests the mediating role of training intention between personality traits and training action planning (*Hypothesis 4a*) and the second one between personality traits and training action initiation (*Hypothesis 4b*). There are significant indirect effects of training intention in relationships between four personality traits and action planning: for extraversion *B* = 0.06, *SEB* = 0.02, 95% CI [0.03; 0.10]; for agreeableness *B* = 0.06, *SEB* = 0.03, 95% CI [0.01; 0.12]; for conscientiousness *B* = 0.07, *SEB* = 0.02, 95% CI [0.03; 0.11], and for openness to experience *B* = 0.11, *SEB* = 0.03, 95% CI [0.05; 0.17], as each of the 95% confidence intervals did not include zero. Only the indirect effect of training intention, when neuroticism is concerned, appeared to be insignificant *B* = -0.03, *SEB* = 0.02, 95% CI [-0.06; 0.01]. However, all indirect effects of personality traits via training plans to training action initiation are insignificant: for extraversion *B* = 0.01, *SEB* = 0.02, 95% CI [-0.01; 0.06]; for agreeableness *B* = 0.02, *SEB* = 0.02, 95% CI [-0.01; 0.11]; for conscientiousness *B* = 0.02, *SEB* = 0.02, 95% CI [-0.01; 0.07]; for openness to change *B* = 0.03, *SEB* = 0.03, 95% CI [-0.01; 0.09], and for neuroticism *B* = -0.004, *SEB* = 0.01, 95% CI [-0.03; 0.01]. These analyses confirm the hypothesis that intention to initiate training mediates between personality traits (except for neuroticism) and training action planning (*Hypothesis 4a*) but not between personality traits and training action initiation (*Hypothesis 4b*).

### Discussion

The results of this study show that openness to experience is important to the initiation of training goal pursuit. This study investigates not only the formulation of intentions and plans, but also actually taking up and completing training activities. The results show that openness to experience plays an important role at all three stages of the process of undertaking training. The effect of this personality trait on action planning is mediated by the training intention. Training intention is also a mediator between three other traits and action planning: extraversion, agreeableness and conscientiousness.

The results also confirmed significant correlations between intention, planning and training action initiation. This is consistent with the results of previous studies concerning goal realization in other domains (Gollwitzer et al., 2008; Gollwitzer and Oettingen, 2012). Planning, however, does not mediate between the intention to undertake training and action initiation. Also training intention does not mediate between personality traits and training action initiation.

## General Discussion

The above studies respond to the gap clearly expressed in the literature: the deficit of research into the role of personality in training initiation (Colquitt et al., 2000; Salas and Cannon-Bowers, 2001; Rowold, 2007). We aim to determine the role of five personality traits at the subsequent stages of the training initiation process, understood as a goal realization process. The results show that, based on personality traits, we may, to some degree, predict training motivation: intention, plan and action initiation. This research is one of the few studies in which not just one but five personality traits were taken into consideration (e.g., Major et al., 2006; Rowold, 2007). The results of both studies described here are highly consistent, despite using different methods of personality traits measurement and different samples. They confirm the role of openness to experience, which is positively related, not only to training intention, but also to action planning and training action initiation. Its relationships with training intention occurred as stronger than with planning of activity (especially in Study 2) and also stronger than with training action. It may be explained by taking into account that these three variables are the successive stages of the process of goal realization (Heckhausen and Gollwitzer, 1987) and personality variables may play more important roles at the initial stages while other motivational variables may be more prominent at later stages (Rauch and Frese, 2007). Greater openness to new situations and curiosity favors the formulation of firm training intentions which, in turn, leads to the formulation of plans and to action initiation (Gollwitzer, 1999; Gollwitzer et al., 2008; Gollwitzer and Oettingen, 2012). These results are consistent with some previous studies on training motivation (Major et al., 2006; Rowold, 2007), offering new evidence of the importance of this personality trait. Openness to experience is manifested by curiosity (McCrae and Costa, 1999), which may cause seeking new opportunities to learn, improve skills, meet new people, and see new places and situations.

In previous studies, attention has mostly been paid to conscientiousness and some research findings (Colquitt and Simmering, 1998; Colquitt et al., 2000; Naquin and Holton, 2002; Major et al., 2006) pointed to the important role of conscientiousness in the prediction of training motivation and motivation to learn; however, other studies did not confirm this (Rowold, 2007). In our research, conscientiousness was not significantly related to either intention or to training initiation directly, but only indirectly to action planning through behavioral intention as a mediator. This may suggest that conscientious people tend to focus on the transfer of knowledge and skills to specific situations at work (Martocchio and Judge, 1997), while this variable is less important in phases initiating the learning process. The results of both our studies regarding agreeableness and extraversion only partly confirm their importance to training initiation understood as the goal realization process. These two traits predict action planning only indirectly by training intention. This adds to a long list of ambiguous evidence (Colquitt et al., 2000) and may suggest that searching for moderators for relationships between conscientiousness or other personality traits and learning, such as, for example, work engagement (Bakker et al., 2012), may help better understand these relationships in future studies. Our results add new empirical evidence, allowing an accumulation of results that could be the basis for new meta-analyses in this area.

The research presented here contributes to the development of the theory of training initiation and to the development of knowledge of goal-directed behavior. The theoretical model we propose explains the process of undertaking training in the context of the theory of goal realization (Heckhausen and Gollwitzer, 1987). This makes it possible to compare studies of undertaking training with the results of studies in other fields, because the theory of goal-directed behavior is widely used in many areas (Armitage and Conner, 2001). The results provide evidence confirming positive correlations between the stages of goal realization processes (intention, planning, and action initiation) in a new domain. So far, many experimental studies have confirmed that declaring an intention to act and planning activities contribute to their undertaking and effective completion (Armitage and Conner, 2001; Gollwitzer et al., 2008; Gollwitzer and Oettingen, 2012). Still, there are few studies that verify these relationships in real life situations and in samples other than student samples. Our studies on undertaking training as a real goal in life, offers a significant contribution to this evidence. Moreover, they were carried out on two independent samples and confirm the goal realization model.

These studies, however, have some limitations. The weaknesses of Study 1 were minimized in Study 2 by including measurement of actual training action; however, both studies are based on self-assessment. They are also based on a theoretical model which emphasizes the planned nature of human actions (Heckhausen and Gollwitzer, 1987) and to a lesser extent takes other factors, such as affect, into account. An analysis of affect and other individual variables, as well as using multilevel study design, which allows analysis of company level characteristics, may be avenues for future studies.

Despite some limitations, the results of these studies offer some practical implications related to human resource development. From an organizational point of view, training is a considerable financial investment and managers would like to offer participation in developmental activities only to those employees who will actually apply the knowledge and skills that they acquire to everyday situations at work. The transfer of knowledge, however, might be, to a great extent, predicted on the basis of motivation to learn (Gegenfurtner and Vauras, 2012). The results of our studies, as well as of others (e.g., Major et al., 2006; Rowold, 2007), indicate that openness to experience is an important determinant of training motivation. For human resource development and management practices it might be doubly important. Firstly, if a given company can offer training only to some employees, the results of personality traits assessment, as well as of the evaluation of training intention and action planning for specific training, may be useful when selecting those who have a greater motivation to learn. This, in turn, will translate into training effectiveness (Tannenbaum et al., 1991). Secondly, the widely used assessment of personality traits during the selection and recruitment of employees may help predict (to some extent) not only their job effectiveness (Judge and Llies, 2002) but also their motivation to learn and participate in training activities. The results of these studies also provide some clues concerning what to pay attention to when trying to improve motivation to participate in training. It is vital to strengthen intentions to undertake developmental actions and encourage employees to plan training activities. Once the intention is formed and the plan developed, an employee is motivated to initiate training.

The studies presented in this article allow us to better understand the factors that may encourage people to learn and develop themselves, which is of substantial social significance. In order to build a knowledge society and to develop skills, it is worth recognizing on what the pursuit of learning in adults may depend.

## Author Contributions

ML was involved in formulating the research question and designing the studies. ML and EP were involved in collecting and analyzing the data, writing the article. Both authors were responsible for drafting and approving the final manuscript.

## Conflict of Interest Statement

The authors declare that the research was conducted in the absence of any commercial or financial relationships that could be construed as a potential conflict of interest.
